# A Novel High Performance Liquid Chromatography Method for Camphor Determination and Application in Cosmetics and Pharmaceuticals: Development and Validation

**DOI:** 10.3390/molecules29184290

**Published:** 2024-09-10

**Authors:** Martin Lalić, Ana Soldić, Zdenka Lalić, Miranda Sertić

**Affiliations:** 1 Apipharma d.o.o., Pharmaceutical-Food, Cosmetic Industry and Trade, J. Kavanjina 26, 10090 Zagreb, Croatia; martin@apipharma.hr (M.L.); zdenka@apipharma.hr (Z.L.); 2 Faculty of Pharmacy and Biochemistry, University of Zagreb, A. Kovačića 1, 10000 Zagreb, Croatia; miranda.sertic@pharma.unizg.hr

**Keywords:** Camphor, HPLC, DAD, internal standard

## Abstract

A novel high-performance liquid chromatography (HPLC) method with 4-*N*,*N*-dimethylaminobenzaldehyde as an internal standard was developed for the determination of Camphor with the main goal of facilitating the analysis of different cosmetic and pharmaceutical products that contain Camphor in analytical laboratories. The method can be applied to cosmetic and pharmaceutical samples such as gels, ointments, and creams containing Camphor. Chromatographic separation was carried out on the Symmetry^®^ C18, 5 μm column (Waters), 250 × 4.6 equipped with guard column E, InertSustain C18, 5 µm, while using the flow of 1.4 mL/min, with a column temperature of 25 °C. The mobile phase consisted of 600 mL of acetonitrile, 400 mL of purified water, and 6 mL of glacial acetic acid. The method was evaluated in accordance with ICH Q2 (R2) guidelines for validation parameters: selectivity, linearity (range 0.10–3.00 mg/mL), the limit of detection (LOD = 0.028 mg/mL), the limit of quantification (LOQ = 0.085 mg/mL), accuracy (confidence intervals < 0.05%), repeatability (peak area ratio = 0.39–1.97), and intermediate precision (peak area ratio = 0.40–1.98). The method is applicable for detecting and quantifying Camphor in a variety of cosmetic and pharmaceutical products from different parts of the world, thus covering the concentrations required by different law legislations.

## 1. Introduction

Cinnamomum Camphora is commonly known as the Camphor tree. It originates from Asia, mainly China, India, South Korea, Japan, Mongolia, and Taiwan but has spread throughout the world over time and can be found in Australia and the United States of America, mainly the state of Florida [1,2]. Camphor is one of the main volatile constituents of Cinnamomum Camphora essential oil [3]. Camphor is a white, crystalline substance with a strong and pungent odor derived from at least a 50-year-old Camphora laurel tree using distillation [3,4,5]. For decades, Camphor was used as a traditional medicine and remedy as a cough suppressant, nasal decongestant, muscle relaxant, and rheumatism reliever [5]. While its use dates far back in history, especially in Asia, research on Camphor also dates back in the past with one of the first, still available papers published in 1894 in the Journal of the Straits Branch of the Royal Asiatic Society stating that Camphor is an important product of Borneo and Sumatra Islands with two distinct products yielded from this highly valued tree, Borneo Camphor, and Camphor oil [6]. In the 1920s, Schoff [7] published his findings on Camphor and the relations of native people of Sumatra, Bali, Borneo, and the Philippines, with the Camphor tree alongside their beliefs and customs regarding the tree. From its purpose in the burial of kings, and earthquake mystical forming crystals to naming Camphor “dragon’s brains”, all of these show the great importance of Camphor among people of Southeast Asia. Camphor has continued to be one of the most highly investigated compounds even today.

Camphor’s effect on human health has been investigated numerous times, and even though it has many positive effects, the toxicity of Camphor has come to attention over the last few years, mostly due to accidental ingestion of Camphorated oil or similar products where most of the patients were children whose outcome was fatal. Fast absorption by ingestion, inhalation, and dermal exposure has been confirmed [4] with a lethal dose of 50 to 500 mg per kg [8]. Typical symptoms of Camphor toxicity after ingestion of a smaller dose include tremors and twitching in the muscles, nausea, vomiting, dizziness, and hallucinations but can progress to cardiac arrhythmias, convulsions, and cardiopulmonary arrest if a large dose is ingested. With a lack of an antidote, the treatment is symptomatic and includes vomiting, which can improve the outcome [8,9,10]. After absorption, Camphor is rapidly distributed throughout the body, including the placental barrier, which is why it is not recommended for use in pregnant women, as the dose that can pass through the placenta is lethal for the unborn baby [4,8,9]. With that in mind, it is important to emphasize that Camphor has a positive effect on human health, which is mostly caused by its strong ability to desensitize sensory nerves by binding on two receptors of the transient receptor potential (TRP) channel superfamily: heat-sensitive TRP vanilloid subtype 1 (TRPV1] and cold-sensitive TRP channel M8 [4,11,12]. It stimulates the analgesic action of capsaicin by desensitizing TRPV1, but even though it has a lower ability to activate TRPV1, it desensitizes it more rapidly than capsaicin, ultimately meaning it leads to faster pain relief than capsaicin [11]. Besides the analgesic effect, the activation of TRPV1 is associated with Camphor counterirritant and antipruritic properties [4]. Camphor also inhibits the TRPA1 receptor and activates TRPM8, whose activation might intensify the effects of menthol when used in products aimed at treating upper respiratory tract infections [12]. Animal studies have shown that Camphor has a significant effect on cough when submitted via nasal application in the form of aromatic vapors on guinea pigs [13], it has the potential to reduce features of fungal pathogenicity [13,14,15], specifically the production of hyphae, and biofilm formation, in concentrations (0.125–0.35 mg/mL) that are non-toxic for porcine liver and therefore may be a promising antifungal agent [14].

Due to its both positive and negative effects on human health Camphor regulation varies between states as can be seen in Table 1. Therefore, the developed method presented in this article was adjusted to fit samples that can be found in the market of Canada, the United States of America (USA), and partially the market of the European Union. The content of Camphor in products, as said in Table 1, within the EU is regulated by Directives and Regulations of the European Parliament and the Council, according to the purpose of the product. According to Regulation (EC) no. 1223/2009 on cosmetic products it is mandatory to declare Camphor whose share exceeds 0.001% for products that are applied to the skin and absorbed and 0.01% for products that are washed off after application to the skin and are not absorbed [16]. The Government of Canada has published a List of Ingredients that are Prohibited for Use in Cosmetic Products [17] where the amount of Camphor is set at a maximum of 3% in the cosmetic products. The content of Camphor in OTC analgesic drugs for external use on the United States market is regulated by Final Tentative Monograph (Over-the-Counter Monograph M017: External Analgesic Drug Products for Over-the-Counter Human Use) under the supervision of the US Food and Drug Administration (FDA) and amounts allowed in this type of product are in the range of 3 to 11% [18].

A few articles on Camphor determination were available via Google Scholar. Our previous article on the simultaneous determination of capsaicinoids and Camphor [19] had a long run of 40 min due to propolis containing an ointment matrix compared to this new method whose run is only 12.5 min, where propolis interfered with compound elution. No other similar HPLC-DAD method for the determination of Camphor was found to be available. Shaikh and Jain [20] published a paper on Curcumin, Piperin, and Camphor determination via HPLC coupled with a UV/Vis detector with detection at 255 nm, which had a lower, but also rather small, linearity range (4–8 µg/mL for Camphor) and had a lower LOD of 0.175 µg/mL and LOQ of 0.531 µg/mL in comparison to our LOD of 0.028 mg/mL (28 μg/mL) and LOQ of 0.085 mg/mL (85 μg/mL). The sample preparation is simple, the accurately weighed sample is dissolved in methanol, and filtered before initiation in the HPLC system. The advantage of our method compared to the method published by Shaikh and Jain is a larger number of different types of samples, given that our method includes gels, ointments, and creams, and their only ayurvedic dental powder. Another HPLC method [21] was found with the use of a refractive index (RI) detector that had a range of 0.32 mg/mL to 1.28 mg/mL and was published in 2012 but without LOD and LOQ values. This method was intended only for topical cream samples and requires a relatively large amount of sample (300 g) for homogenization, after which 1 g of sample is diluted in 25 mL of methanol, dissolved, filtered through Whatman filter paper No. 1, and after that through a 0.45 μm syringe filter.

The method presented in this article is the only one with an internal standard that minimizes the effects of random and systematic errors during analysis and helps to improve the precision of results. Other methods such as gas chromatography with a flame ionization detector (GC-FID) [22] and the ultra-high-performance supercritical fluid chromatographic method (UHPSFC) [23] are available for implementation for those with the right equipment.

## 2. Results

### 2.1. Method Development

**Figure 1:**
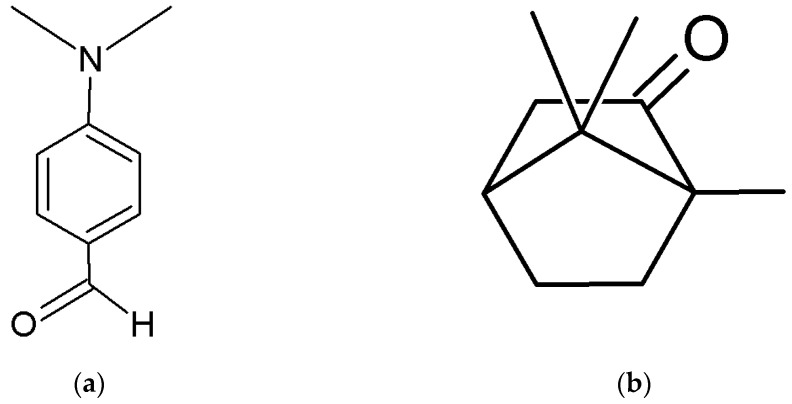
Chemical structure of (**a**) 4-*N*,*N*-dimethylamino benzaldehyde (ISTD) and (**b**) Camphor.

**Figure 2:**
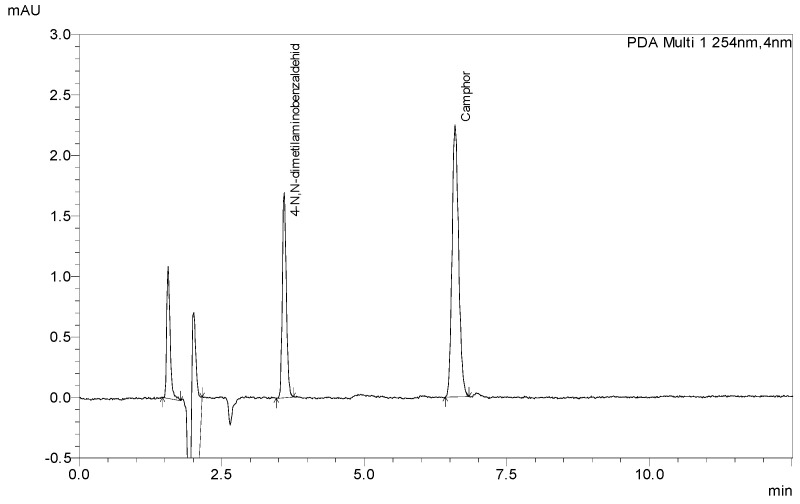
The chromatogram of standard solutions of ISTD (1 µg/mL) and Camphor (1000 µg/mL).

**Figure 3:**
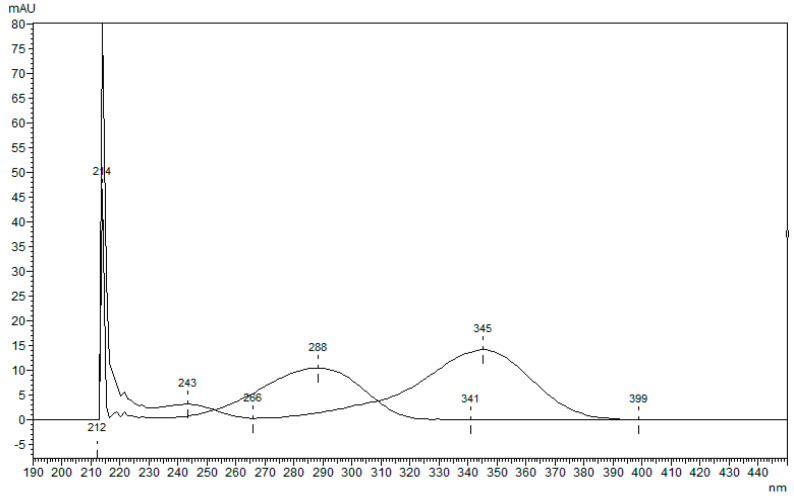
The overlaid spectrum of ISTD (345 nm) and Camphor (288 nm) at concentrations 200, 500, and 1000 µg/mL.

The lack of analytical methods for the determination of Camphor via HPLC-DAD led us to the development of one due to the high demand for products for pain relief containing Camphor and the low possibilities of quality control in such products. The newly developed method with 4-*N*,*N*-dimethylamino benzaldehyde as an internal standard (ISTD) was used to determine the content of Camphor in laboratory-prepared samples and store-bought samples. ISTD was chosen as suitable after being compared to Camphor by its chemical structure (Figure 1), retention time (Figure 2), and overlaid spectrum (Figure 3) of ISTD and Camphor in different concentrations (0.20, 0.50, and 1.00 mg/mL).

For the purpose of validation and verification of the newly developed method, three groups of possible products containing Camphor, gel, cream, and ointment were laboratory prepared. Camphor standard and internal standard were added to all products in the same known concentration after which the extraction and analysis were conducted.

### 2.2. Optimization of the Extraction of Camphor

Multiple extractions with different conditions and different ratios of solvents were conducted and results were recorded (Supplementary Materials). All samples used for the optimization of the extraction of Camphor were laboratory-prepared gels, creams, and ointments. ISTD was added to each sample before the beginning of the extraction and was homogenized with the sample. All analyses were made in triplicate. Conditions of extraction included different temperatures (35 or 50 °C), application of ultrasound or not, solvent ratio (96% ethanol: mobile phase), and duration of the extraction (15 or 30 min) (Supplementary Materials). Extractions with the highest recovery (Formula (1)) values (Table 2) were selected and samples were prepared according to those conditions (selected preparations are described in detail in the Sample Preparation Section). As seen in Table 2, cream had the highest extraction recovery at 99.17 ± 0.76%, followed by ointment at 98.72 ± 0.36%, and the gel had the lowest extraction recovery at 95.83 ± 0.61%. It was shown that ultrasound yields better recovery results for ointment and gel, but not for cream. A shorter amount of time was suitable for samples of gel and cream, while a longer time suited the ointment samples. The ointment was the only sample that gave better results at higher temperatures, which could be caused by the matrix nature since vaseline and lanoline are easier to melt at higher temperatures, which could lead to easier extraction of Camphor. As for the solvent selection, ointment, and cream samples gave better recovery results with only 96% ethanol, while gel gave the best result when using the 3:2 ratio of solvents.

Formula (1). Recovery was calculated using the following formula:

(1) $$\%\;Recovery=\frac{found\;concentration}{added\;concentration\;}\times 100$$

Table 3 presents recovery results for other laboratory-prepared samples that yielded a recovery of over 90%. From this, it is easy to conclude that the ointment extraction does not depend on using the ultrasound, but rather on a higher temperature, longer time, and using only 96% ethanol as solvent. Gel samples gave good extraction results regardless of the time of the extraction, but only with a 3:2 ratio of extraction solvents at a temperature of 35 °C, and the use of ultrasound was shown to be beneficial. This can be seen in the example of the last gel sample that had the lowest recovery value even though it had almost all of the mentioned conditions but had not been extracted with the ultrasound. Cream samples showed better recovery values when a 5:0 solvent ratio was used at a temperature of 35 °C with little to no dependence on time or use of the ultrasound. On the other hand, a higher content of mobile phase in the extraction solvent of cream and ointment led to blurred samples event after filtering samples through 0.45 µm PA filters, while a higher content of 96% ethanol in the case of gels led to gelation of sample in vial after remaining at room temperature for more than 10 min. These samples were not analyzed due to the possibility of pollution of the HPLC column, but their extraction conditions are shown in the Supplementary Materials along with extraction conditions and recovery values of all the samples with recovery values below 90%.

### 2.3. Method Validation

Validation was conducted by performing system suitability, selectivity, linearity range, the limit of detection (LOD), the limit of quantification (LOQ), accuracy, repeatability, and intermediate precision of measurement of peak area ratio and retention time ratio following the current ICH Q2 (R2) guidelines (2020) [24].

The selectivity of the method was studied by measurement of peak purity using the LabSolution softwar, version 5.32 SP1. Peak purity evaluation was performed to ensure that no co-migration substance contributed to the response of the peaks. The linearity range was evaluated by initiating a series of 5 standard solutions for each concentration level, 0.10–3.00 mg/mL. The linear concentration range, LOD, and LOQ are summarized in Table 4 and Table 5. LOD and LOQ were calculated based on the standard deviation of the response and the slope following the equations

LOD = 3 Q/S

LOQ = 10 Q/S

where S is the calibration curve’s slope and Q is the response’s standard deviation of peak area ratio. The developed method has suitable sensitivity, as demonstrated by the obtained data, and allows its use in a pharmaceutical and cosmetic product.

Repeatability (intra-day) and intermediate (inter-day) precision data were used to compute precision (Table 6) and accuracy (CI = 95%). Multiple injections (*n* = 5) of the standard solutions of three concentration levels (0.20, 0.50, and 1.00 mg/mL) were used to measure intra-day precision by observing changes in peak area ratio and retention times ratio. Low RSD values indicate that the method’s repeatability is satisfactory. Over three days, the inter-day precision was assessed. Newly made standard solutions were used every day. Every day, five injections of these solutions were assessed. The internal standard, Camphor retention time ratio, and peak area ratio were calculated, and both RSD values showed that the intermediate precision is appropriate. Additionally, accuracy was tested by performing the spiking of the three (one for each group) randomly chosen store-bought samples. Samples were spiked with three different concentrations of 0.50, 1.25, and 2.50 mg/g samples, which proved that the method has good accuracy, recovery, and RSD values. Results are shown in Table 7.

The column’s temperature and wavelength were adjusted throughout the robustness test. Triplicate analyses were carried out at one concentration level (1.50 mg/mL) and three different wavelengths: 250, 254, and 260 nm, and different column temperatures, 20 °C and 35 °C. The results indicated that the procedure was determined to be reliable, with a peak area ratio of 3.08 ± 0.07%, and a retention time area ratio of 1.83 ± 0.00%.

### 2.4. Application of the Method

**Figure 4:**
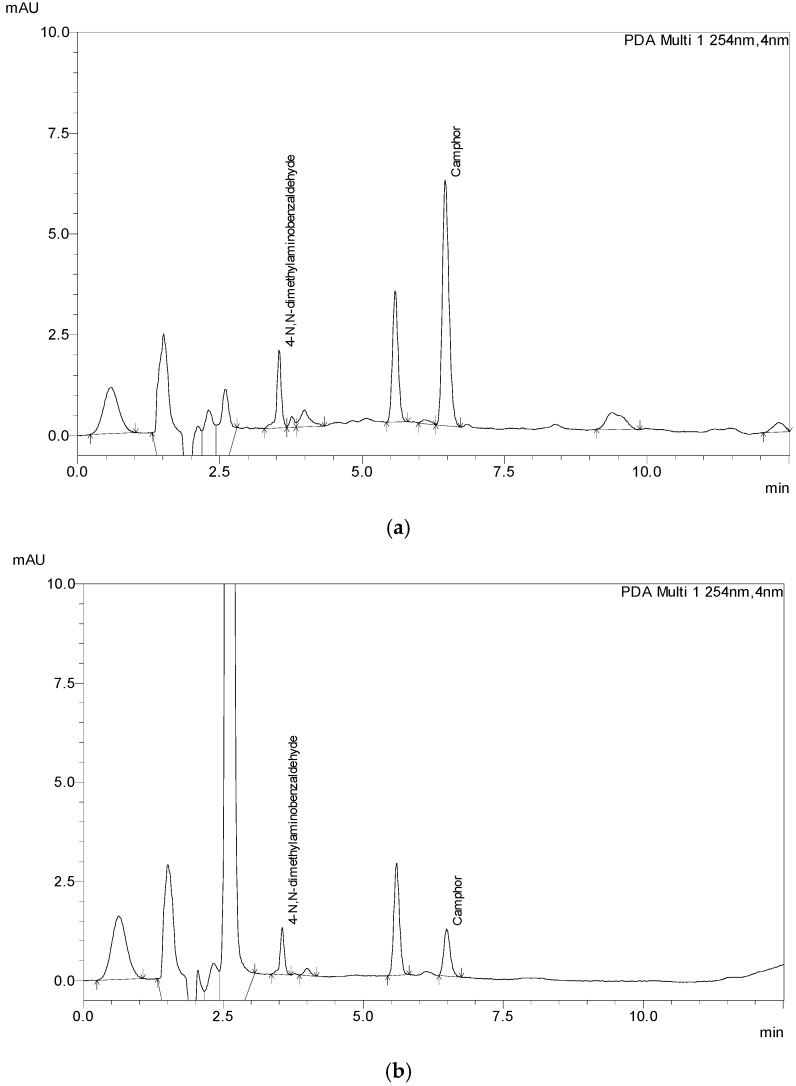

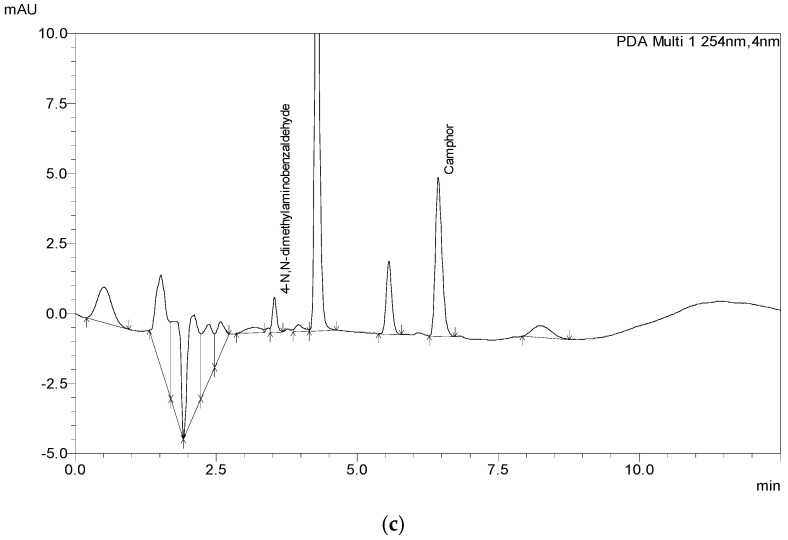
The chromatograms of store-bought samples: (**a**) ointment sample (S10), (**b**) gel sample (S4), (**c**) cream sample (S13).

To test the applicability of the method, 13 samples were acquired from different markets, including the EU, US, and Canada markets. Samples were prepared and extracted the same way as the laboratory-made samples (Figure 4) depending on what category they belonged to, six samples of gel, three samples of cream, three samples of ointment, and one spray sample. Given the fact that most of the samples were cosmetic products, none of the products had declared the amount of Camphor in its composition on its label, not even the OTC samples. Thus, the analyses were performed with 1 g of sample. After the initial results sample mass and the volume of the extraction solution were adjusted to fit the method and the analysis was conducted again, with sample mass in the range of 0.2 g to 5 g of sample (5000 μL for spray sample). Additionally, each sample was spiked with 1.15 mg of Camphor/g sample to test the applicability of the method. All analyses were performed in triplicate. The results are given in Table 8, and chromatograms of the samples are shown in Figure 4.

## 3. Discussion

The purpose of this study was to explore and develop a method for the determination of Camphor in a complex matrix such as pharmaceuticals and cosmetics. Due to differences between matrixes that include this group of products, easy and fast sample preparation alongside agile and simple analytical methods was needed to fit the requirements of quality control for the industry. The wide linearity range (0.1 to 3.00 mg/mL) corresponds with the legal requirements of the USA and Canada without demanding great dilutions of samples, which leads to a lower chance of analytical errors during the procedure but also during the latter calculation. The regulations of the EU require declaration of the Camphor on the label if its concentration exceeds 0.01% (0.1 mg/g) for products that are meant to be washed off the skin and 0.001% (0.01 mg/g) for those that are meant to be absorbed. Thus, the method can be used for products with a declared amount of Camphor of 0.1% or more; for lower concentrations, additional method development should be conducted. While testing store-bought samples, we did not come across a sample with such a low concentration’ in other words, all the samples that had Camphor declared had a concentration above 0.62 mg/g of sample, which was the lowest concentration found among samples. The analyzed store-bought samples have a wide range of Camphor concentrations, starting from 0.62 mg/g (0.062%) of sample in one sample up to 105.7 mg/g (10.57%) in another. The fast run of 12.5 min provides a greater number of samples in a short period of time. No expensive equipment or chemicals are needed to perform the analysis, and thus, it can be used as a quick in-process control during the research and development of new products or the production of existing products. It has been tested on samples such as gels, creams, ointment, and even on spray, but it could be easily used on other samples containing Camphor such as shampoos or lotions.

## 4. Materials and Methods

### 4.1. HPLC Instrumentation and Chromatographic Conditions

The Shimadzu Prominence HPLC system (Kyoto, Japan), which consists of an autosampler (SIL20-ACX), two isocratic pumps (LC-20ADXR), a column chamber (CTO-20AC), a diode array detector (SPD-M20A), and LabSolution software, version 5.32 SP1 was used to analyze the samples. A Symmetry^®^ C18, 5 μm column (Waters, Milford, MA, USA), 250 × 4.6 mm, equipped with Guard Column E InertSustain C18, 5 µm, was used to achieve the component separation.

### 4.2. Chemicals and Reagents

Camphor (≥97.5%) was purchased from Sigma-Aldrich (St. Louis, MO, USA), 4-*N*,*N*-dimethylaminobenzaldehyde was purchased from Kemika (Zagreb, Croatia), Acetic acid (glacial) 100% was obtained from Merck (Darmstadt, Germany), J.T. Baker HPLC grade acetonitrile was purchased from Avantor (Gliwice, Poland), ethanol 96% from Fagron (Donja Zelina, Croatia), and ultrapure water was obtained via the Letzner water purification system (Hückeswagen, Germany).

### 4.3. Preparation of Standard Solutions

The stock solution of Camphor was prepared by dissolving 100 mg in 10 mL of 96% ethanol in order to obtain a final concentration of 10 mg/mL. ISTD was prepared by dissolving 10 mg in 100 mL of 96% ethanol. These solutions were stored at −18 °C with temperature monitoring using the Data Logger LOG200 purchased from Dostmann Electronic GmbH (Reicholzheim, Germany). Calibration standards were prepared with mobile phase in the following concentrations: 0.10, 0.20, 0.50, 1.00, 2.00, and 3.00 mg/mL each with 0.001 mg/mL of ISTD.

### 4.4. Sample Preparation

The cream was prepared by using a commercially available base named Belobaza^®^ (Belupo, Croatia), which contains ultrapure water, vaseline, cetostearyl alcohol, liquid paraffin, ceteareth-20, benzyl alcohol, sodium phosphate, phosphoric acid, sodium hydroxide. The gel base was prepared by mixing ultrapure water, glycerol, sodium hydroxide, carbomer 940, sodium benzoate, and potassium sorbate. The ointment base was made by mixing vaseline and lanoline. To each of these bases, the exact amount of Camphor standard was added, resulting in a product that contains 1.50 mg of Camphor/g of the finished product.

### 4.5. Preparation of the Samples for the Extraction

- Cream

For the cream analysis, 1 g of the cream sample was accurately weighed in a 15 mL extraction tube in which 9.9 mL of 96% ethanol and 100 µL of ISTD (concentration of 0.001 mg/mL) were added. The sample was extracted for 15 min in the water bath at 35 °C with occasional stirring without ultrasound.

- Gel

For the gel analysis, 1 g of the gel sample was accurately weighed in a 15 mL extraction tube in which 9.9 mL of a mixture of solvents, in ratio 3:2 of 96% ethanol/mobile phase, and 100 µL of ISTD (concentration of 0.001 mg/mL) were added. The sample was extracted for 15 min in the water bath at 35 °C with occasional stirring with the use of the ultrasound.

- Ointment

An amount of 1 g of ointment was accurately weighed in a 15 mL extraction tube in which 9.9 mL of 96% ethanol and 100 µL of ISTD (concentration of 0.001 mg/mL) were added. The sample was extracted for 15 min in the ultrasound water bath at 50 °C with occasional stirring, without the use of the ultrasound.

After cooling the sample, the sample was then centrifuged for 5 min at 3500 rpm. The supernatant was filtered through a 0.45 µm nylon filter and initiated into the system.

### 4.6. Mobile Phase and HPLC Analysis

The mobile phase containing acetonitrile, ultrapure water, and glacial acetic acid in a ratio of 600:400:6 mL was degassed with a vacuum pump before use. The flow of the mobile phase was 1.4 mL/min at a column temperature of 25 °C with an injection volume of 10 μL. Spectrum recording was performed in the wavelength range from 190 to 370 nm, while detection was performed at a wavelength of 254 nm. The run was 12.5 min long, with the relative retention times of Camphor 6.605 min and internal standard 3.590 min. The identification of Camphor was performed by comparing the retention times of standard solutions and sample solutions and unquestionable identification was confirmed by comparing the specific spectrum of standard solutions (Camphor and ISTD) and sample solutions. Quantification was performed by LabSolution software, version 5.32 SP1 by comparing the peak area ratio of the internal standard and Camphor standard.

## 5. Conclusions

The isocratic HPLC method coupled with a DAD detector for the determination of Camphor was developed with the specific aim of detection and quantification of Camphor in pharmaceuticals and cosmetics. Due to the different law requirements in different markets, this method applies to Canada, the USA, and part of the EU market (for products that are washed off after application to the skin and are not absorbed) with correct sample mass adjustment. What is important to emphasize is that, unlike the Canada and USA, the EU does not have a minimum or maximum concentration allowed in products, but only limits above which concentration Camphor must be declared on the label. The production of different pharmaceutical and cosmetic products with Camphor (for different markets) requires control of the Camphor content in them. In the absence of HPLC methods for the determination and quantification of Camphor in such products, this method with an internal standard was developed that enables the quantification of Camphor in non-prescription drugs and cosmetic products within the range determined by the specified legal regulations (the EU, Canada, and the USA). The method applies to the pharmaceutical and cosmetic industry for quality control of finished products and stability control of products with Camphor.

## Figures and Tables

**Table 1:** Camphor regulation in the EU, Canada, and the USA.

Country/Region	Max Concentration	Type of Product
EU [16]	Not specified on the EU level Must be declared if it exceeds − 0.001% for products that are applied to the skin and absorbed − 0.01% for products that are washed off after application to the skin and are not absorbed	Cosmetics
Canada [17]	3%	Cosmetics
USA [18]	3–11%	Over-the-counter drug (OTC)

**Table 2:** Extraction recovery of laboratory-prepared samples with the highest recovery values for each group of samples.

Sample Type	Sample Mark	Extraction Conditions				Recovery (%)	RSD (%)
		Solvent Ratio (96% EtOH:MP *)	Temperature (°C)	Time (min)	Ultrasound (Yes or No)		
Cream	K2	5:0	35	15	No	99.17	0.76
Ointment	M5	5:0	50	15	Yes	98.72	0.36
Gel	G17	3:2	35	15	Yes	95.83	0.61

* MP—Mobile Phase (acetonitrile/ultrapure water/glacial acetic acid = 600:400:6 mL).

**Table 3:** Extraction recovery of other laboratory-prepared samples with extraction values over 90%.

Sample Type	Sample Mark	Extraction Conditions				Recovery (%)	RSD (%)
		96% EtOH:MP (mL)	Temperature (°C)	Time (min)	Ultrasound		
Gel	G19	3:2	35	30	Yes	93.27	1.54
Gel	G20	3:2	35	30	No	90.62	1.78
Ointment	M8	5:0	50	30	No	94.35	1.13
Ointment	M7	5:0	50	30	Yes	93.65	1.71
Ointment	M6	5:0	50	15	No	90.47	1.92
Cream	K3	5:0	35	30	Yes	95.83	0.61
Cream	K4	5:0	35	30	No	91.62	1.30
Cream	K17	3:2	35	15	Yes	91.39	1.94

**Table 4:** Linearity range and regression equation for Camphor determination.

Linearity Range (mg/mL)	Regression Equation	R
0.10–3.00	y = 0.00208x − 0.00537	0.999

**Table 5:** Limits of detection and quantification for Camphor analysis.

LOD (mg/mL)	LOQ (mg/mL)
0.028	0.085

**Table 6:** Precision and accuracy data for Camphor (0.20, 0.50, and 1.00 mg/mL) analysis.

Intra-Day Precision (*n* = 5)						
	0.20 mg/mL		0.50 mg/mL		1.00 mg/mL	
	Peak Area Ratio	Retention Time Ratio	Peak Area Ratio	Retention Time Ratio	Peak Area Ratio	Retention Time Ratio
**Average**	0.39	1.83	0.98	1.83	1.97	1.83
**RSD (%)**	0.85	0.05	0.65	0.08	0.54	0.04
**CI (** **<0.05%)**	0.004	0.001	0.008	0.002	0.015	0.000
**Inter-Day Precision (*n* = 15)**						
	**0.20 mg/mL**		**0.50 mg/mL**		**1.00 mg/mL**	
	**Peak Area** **Ratio**	**Retention Time Ratio**	**Peak Area** **Ratio**	**Retention Time Ratio**	**Peak Area** **Ratio**	**Retention Time Ratio**
**Average**	0.40	1.82	0.99	1.82	1.98	1.83
**RSD (%)**	0.94	0.04	0.47	0.08	0.98	0.09

**Table 7:** Recovery and RSD values of spiked store-bought samples.

Sample Mark	0.50 mg/g of Sample		1.25 mg/g of Sample		2.50 mg/g of Sample	
	Recovery (%)	RSD (%)	Recovery (%)	RSD (%)	Recovery (%)	RSD (%)
**S2**	97.95	0.15	98.26	0.44	98.08	0.68
**S5**	94.81	0.71	93.73	0.58	94.21	0.69
**S10**	97.69	0.52	97.49	0.62	97.58	0.50

**Table 8:** Camphor concentration and recovery values of store-bought samples.

Sample Mark	Sample Type	Category	Market in Which Sample Is Available	Concentration of Camphor (mg/g of Sample)	Recovery (%)	RSD (%)
S1	ointment	cosmetics	EU	0.71	86.14	1.13
S2	gel	cosmetics	EU	1.24	100.99	0.74
S3	gel	OTC medication	USA/Canada	21.57	98.26	0.77
S4	gel	cosmetics	EU	6.98	86.47	0.14
S5	cream	cosmetics	EU	9.21	87.73	1.44
S6	gel	cosmetics	EU	0.87	92.14	3.34
S7	spray	cosmetics	EU	3.9	89.78	0.19
S8	ointment	cosmetics	USA	105.7	97.49	0.29
S9	gel	cosmetics	EU	12.89	93.82	0.09
S10	ointment	OTC medication	USA	38.25	85.19	2.40
S11	cream	cosmetics	EU	0.99	104.60	5.38
S12	gel	cosmetics	EU	0.62	96.42	0.02
S13	cream	cosmetics	USA/Canada	98.16	89.37	1.13

## Data Availability

All supporting data can be obtained from the corresponding author upon formal request.

## Supplementary Materials

The following supporting information can be downloaded at: https://www.mdpi.com/article/10.3390/molecules29184290/s1, Extraction conditions and recoveries.
